# Impact of ontogeny and spines on the hydrodynamic performance of the Cambrian arthropod *Isoxys*

**DOI:** 10.1098/rsos.240894

**Published:** 2024-12-11

**Authors:** Stephen Pates, Jiaxin Ma, Yu Wu, Dongjing Fu

**Affiliations:** ^1^ Department of Zoology, University of Cambridge, Cambridge CB2 3EJ, UK; ^2^ Centre for Ecology and Conservation, University of Exeter, Penryn Campus, Penryn TR10 9FE, UK; ^3^ Shaanxi Key Laboratory of Early Life and Environments, State Key Laboratory of Continental Dynamics, and Department of Geology, Northwest University, Xi’an 710069, People’s Republic of China; ^4^ Institute of Earth Sciences, University of Lausanne, Lausanne, 1015, Switzerland

**Keywords:** Cambrian explosion, allometric growth, computational fluid dynamics, zooplankton, pelagic

## Abstract

A metazoan-dominated biological pump was established early in the Phanerozoic, a time that saw the evolution of the first pelagic euarthropod zooplankton such as some species of the Cambrian bivalved euarthropod *Isoxys*. Pelagic groups evolved from benthic stock, in many cases through neoteny and retention of characteristics from planktic larval stages. However, *Isoxys* brooded eggs and did not have a planktic larval stage, precluding this route into the pelagic realm. Computational fluid dynamics was used to quantify hydrodynamic performance through the ontogeny of two hyperbenthic species of *Isoxys, Isoxys auritus* and *Isoxys minor*. Coefficients were used to quantify forces for different carapace shapes over a range of biologically relevant sizes and swimming speeds. Streamlining and lift generation were greater for later growth stages, a consequence of carapace asymmetry and elongated anterior and posterior spines. Simulations performed with anterior spines artificially removed demonstrate the importance of this feature for lift generation, with a minimal impact on drag. Elongated spines and faster swimming can also be considered anti-predatory, and the reduction of drag would have reduced the detectability of *Isoxys* to predators. Taken together, it is likely that pelagic *Isoxys* species evolved from benthic stock through the co-option of anti-predatory features.

## 1. Introduction

The fossil record documents a rapid increase in organismal diversity, disparity and body size [1–4] alongside the convergent development of disparate predatory and defensive hard tissues and skeletons in many metazoan groups [5–8] during the early Palaeozoic. This increase in animal diversity contributed to nested feedback loops of the Earth system, developmental and ecological processes, generating cascading effects [9,10], including the establishment of tiered trophic structures that transformed the biosphere from a microbial world driven by physical processes to one engineered by metazoans [9,11,12]. Coevolutionary processes drove changes in food web complexity, body size and behaviour through the early Palaeozoic radiation and beyond, with profound impacts on Earth systems, ecosystem stability and evolutionary feedback [9,11–20].

Critical for the support of a diverse array of life in Cambrian bottom water communities was the establishment of complex food webs and a metazoan-dominated biosphere [11,12,21,22]. The establishment of an animal-dominated biological pump revolutionized the transfer of nutrients, energy and biomass in the oceans, increasing flux from the pelagic to the benthic realm and oxygenated bottom waters [12,18]. Pelagic macrozooplankton, which can clear surface waters by feeding on primary producers, contribute larger carcasses and waste products to sinking aggregates and move vertically in the water column forming an important constituent of this pump in the modern ocean [23]. These first appear in the fossil record in the Cambrian [24] and may have also driven size increases in phytoplankton [22,25–27].

*Isoxys* was an abundant representative in Cambrian ecosystems [28–30] with species filling a range of important ecological roles across benthic and pelagic realms, including as visual predators and vertically mobile euarthropod zooplankton [26,29,31–35]. The carapace not only played a hydrodynamic role [26,31] but also was important for the defence and protection of brooded eggs in at least some species, with anterior and posterior spines possibly playing a defensive role [30,31]. An ecological shift from the benthic to pelagic realm could have been induced by abiotic factors (e.g. low oxygen or anoxia in bottom waters), biotic pull (positive trophic and nutrient incentives) or biotic push (predator escape) factors. Pelagic zooplankton has evolved numerous times from benthic ancestors [36], often facilitated through heterochrony, specifically neoteny, with larval characters retained [27,37]. However, as *Isoxys* brooded eggs and did not have a planktonic larval stage [30], this mechanism was not possible for the genus.

Here we explore the impact of increased size and allometry on the function of the carapace in the bivalved stem-group euarthropod *Isoxys,* specifically two hyperbenthic taxa *Isoxys auritus* [38] (Chengjiang) and *Isoxys minor* [30] (Qingjiang and Guanshan). The detailed descriptions of the ontogeny in these two species provide the opportunity to assess the changing hydrodynamic performance of the carapace with size and provide an example of how anti-predatory characteristics might have been coopted for lift generation during ontogeny, facilitating the evolution of a pelagic life mode from benthic stock.

## 2. Methods

Carapace outlines of the smallest, intermediate and largest representatives of two species of *Isoxys* with described ontogenetic sequences were constructed using Inkscape 1.0. For *I. auritus*, these outlines represent carapaces 5, 25 and 45 mm in length, including both morph A and B in the larger size classes [39], while for *I. minor* outlines represent carapaces 10, 15 and 25 mm in length [30]. Outlines were imported into the R environment, scaled, centred and sampled at 64-point resolution using functions from the *Momocs* package [40,41]. Coordinates were then exported into .txt format using a function (provided in the supplementary information of [26]) suitable for import into ANSYS Workbench 2021 R2 and 2024 R2 for computational fluid dynamics (CFD) simulations.

**Figure 1.**
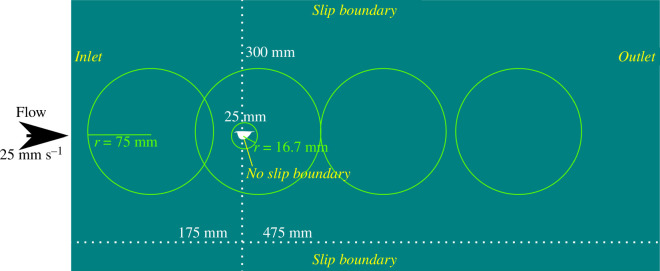
Experiment set-up for CFD simulations. Green circles indicate spheres of influence of radius *r* created during the meshing phase. Exact parameters can be gathered from archived workbench projects, which are provided in the accompanying OSF project. Further details for validation and verification of this set-up can be found in the OSF project accompanying a previous study exploring the hydrodynamics of *Isoxys* [26], https://osf.io/yvenb.

Two-dimensional simulations of laminar flow around the carapaces were undertaken. A two-dimensional area 650 mm in length and 300 mm in height was created, with the carapace positioned 175 mm from the inlet and 150 mm from both upper and lower boundaries (figure 1). During the meshing step, four spheres of influence were created, in order to capture the details of the wake behind the carapace. A further smaller sphere of influence in the area immediately around the carapace served to provide high-resolution detail of the disturbance to the flow in this area (figure 1). A further additional meshing control, a face sizing, was applied to the margin of the carapace itself. A zero-pressure control was applied to the outlet, while the upper and lower boundaries were treated as slip boundaries. The carapace itself was treated as a no-slip boundary. This simulation set-up followed that of a previous study on the hydrodynamic performance of *Isoxys* species [26]. As part of this previous study, simulated results from this set-up were compared to data from NACA 4402, NACA 4404 and NACA 4702 airfoils, at a Reynolds number of 1000 [42], showing that it performs well at low Reynolds numbers, particularly for broad airfoil shapes. However, currently, no suitable data are available for verifying the performance of this simulation set-up at the lowest Reynolds numbers (as low as 13.5) in this study.

Carapaces were standardized to a dorsal length of 25 mm. This allows the performance of the carapace shape to be assessed independently of its length. CFD simulations were conducted using the steady-state laminar solver in ANSYS Fluent (Ansys Academic, Release 2021 R1). This model performed best at low Reynolds numbers (compared to shear-stress transport and k-epsilon models; see the supplementary materials 2 of [26]). Simulations were considered converged when residuals were less than 10^−6^.

Carapaces were orientated at angles of attack from −3 to +3 degrees, at one-degree increments, to find the angle of attack with the lowest drag. A range of flow speeds was then applied to quantify hydrodynamic performance across Reynolds numbers (tables 1 and 2) reflecting size increases during growth observed from fossil material [30,38], and swimming speeds (in body lengths per second) previously reported for swimming euarthropods [43]. When solutions did not converge at a Reynolds number for an individual outline, this Reynolds number was treated as indicating the transition from laminar to turbulent flow. No results were reported for that simulation, and no higher Reynolds number simulations were performed for that carapace shape.

**Table 1.** Calculation of biologically relevant Reynolds numbers for CFD simulations. (BL = body length; Re = Reynolds number.)

valve length (m)	velocity (BLs^−1^)	velocity (ms^−1^)	Re
0.005	1	0.005	13.59788
0.005	2	0.01	27.19577
0.005	3	0.015	40.79365
0.005	5	0.025	67.98942
0.005	8	0.04	108.7831
0.005	10	0.05	135.9788
0.025	0.5	0.0125	169.9735
0.025	0.7	0.0175	237.963
0.025	0.75	0.01875	254.9603
0.025	1	0.025	339.9471
0.025	1.25	0.03125	424.9339
0.025	1.5	0.0375	509.9206
0.045	0.5	0.0225	550.7143
0.045	0.57	0.02565	627.8143
0.045	0.75	0.03375	826.0714
0.045	1	0.045	1101.429

**Table 2.** Calculations for velocity required to simulate that Reynolds number for a carapace length of 25 mm, and values input into ANSYS Fluent. (Re = Reynolds number.)

valve length (m)	Re desired	velocity required (ms^−1^)	velocity input (ms^−1^) (3sf)
0.025	13.5	0.000992802	0.000993
0.025	27	0.001985603	0.00199
0.025	40	0.002941634	0.00294
0.025	70	0.00514786	0.00515
0.025	110	0.008089494	0.00809
0.025	135	0.009928016	0.00993
0.025	170	0.012501946	0.0125
0.025	255	0.018752918	0.0188
0.025	340	0.025003891	0.025
0.025	425	0.031254864	0.0313
0.025	510	0.037505837	0.0375
0.025	550	0.040447471	0.0404
0.025	625	0.045963035	0.046
0.025	700	0.051478599	0.515
0.025	825	0.060671206	0.0607

Further analyses were performed on four carapace shapes representing the two *I. auritus* morphs at different sizes (25 and 45 mm total length) but with anterior and posterior spines shortened. The proportions and length between the spines were controlled to be the same for the corresponding shape with spines, meaning that the total length of these carapace shapes was less than 25 mm. The same inlet velocities and orientation in the flow were used for the carapaces with spines. Thus, for these simulations, the Reynolds numbers were slightly lower for each experiment compared to the corresponding carapace shape with spines. These shapes facilitated exploration of the impact of the spines on the drag and lift of these carapaces.

Coefficients of drag and lift (*C*_*d*_, *C*_*l*_) were calculated for each converged simulation. Coefficients of drag and lift are proportional to the force (either drag or lift, *F*_*d*_ and *F*_*l*_, respectively), inverse square of swimming speed (*u*) and the inverse of the reference length (*A*) (equations (2.1) and (2.2); *ρ* is the density of the fluid):


(2.1) $$C_{d}=\;\frac{2F_{d}}{\rho u^{2}A},$$

(2.2) $$C_{l}=\;\frac{2F_{l}}{\rho u^{2}A}.$$

These dimensionless quantities are specific to individual Reynolds numbers for a given shape. This means that the drag and lift force of carapaces of different shapes and sizes at a range of swimming speeds can then be calculated, from a given Reynolds number, using equations (2.1) and (2.2). These coefficients, and the maximum Reynolds number at which simulations were converged, were used to calculate the maximum velocity (in ms^−1^ and body lengths s^−1^) of each carapace before transition to turbulent flow.

Drag coefficients at intermediate Reynolds numbers were used to compare the absolute drag forces experienced by different carapace shapes, with the length of the carapace between the spines (rather than the total length including the spines) kept constant. This required comparing shapes at different Reynolds numbers (tables 2 and 3). In order to calculate the drag and lift forces for Reynolds numbers intermediate of those simulated, the lm function in R (stats package; [40]) was used to fit a model using the following formula:


(2.3) $$C_{d}=0+I*\;\sqrt{\frac{1}{Re}}.$$

**Table 3.** Calculations of Reynolds number required to compare to *I. minor* and *I. auritus* 5 mm morphs, keeping the length of carapace between the spines constant, for Reynolds numbers 13.5, 68 and 170, respectively.

morph	valve length (m)	velocity (ms^−1^)	Reynolds number
*I. auritus* A, 25 mm	0.0056	0.005	15.2
*I. auritus* A*,* 45 mm	0.0061	0.005	16.5
*I. auritus* B, 25 mm	0.0053	0.005	14.5
*I. auritus* B, 45 mm	0.0064	0.005	17.4
*I. auritus* A, 25 mm	0.0056	0.025	76.05
*I. auritus* A*,* 45 mm	0.0061	0.025	82.9
*I. auritus* B, 25 mm	0.0053	0.025	72.3
*I. auritus* B, 45 mm	0.0064	0.025	87.2
*I. auritus* A, 25 mm	0.028	0.0125	190
*I. auritus* A*,* 45 mm	0.031	0.0125	207
*I. auritus* B, 25 mm	0.027	0.0125	181
*I. auritus* B, 45 mm	0.032	0.0125	218

The coefficient *I* returned by each model was used to calculate a drag coefficient *C*_*d*_ for a given Reynolds number, Re, for a given carapace shape. Further details of the linear models are provided in the electronic supplementary material.

## 3. Results

**Figure 2.**
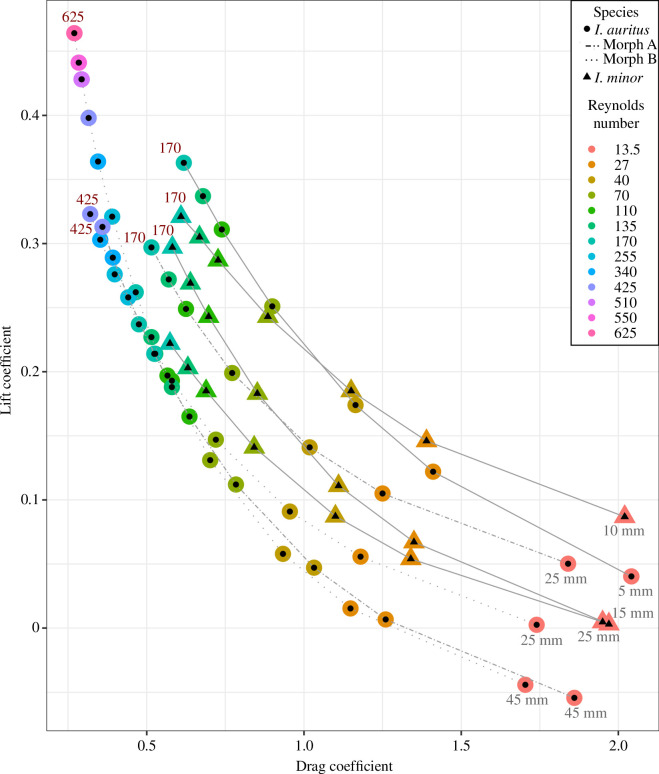
Plot of drag coefficients against lift coefficients for each carapace shape and Reynolds number. Lines join results from a single carapace shape at multiple Reynolds numbers. Grey labels on right-hand side indicate size of carapace of the fossil from which the shape was created. Maximum Reynolds number is given in red in the top left.

**Figure 3.**
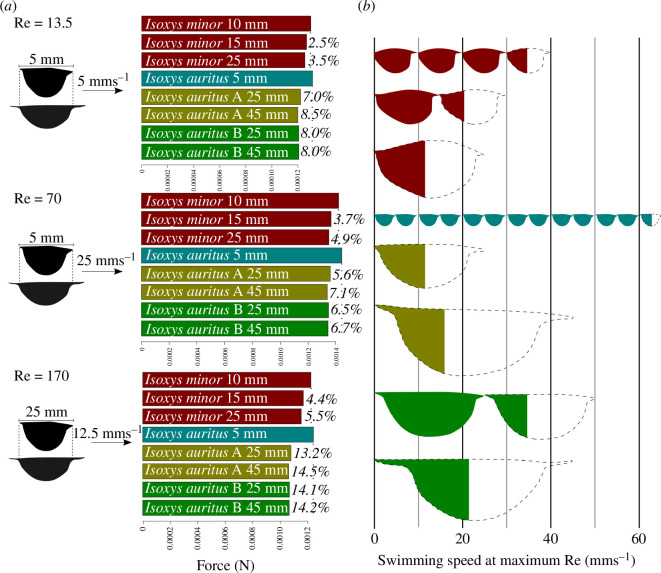
Relationship between size, shape, drag force and hypothesized swimming speed in *Isoxys*. (*a*) Drag forces for carapaces calculated from drag coefficients using equation (2.1) for three representative lengths and swimming speeds for *Isoxys:* carapaces of 5 mm length travelling at one and five body lengths per second, and carapaces of 25 mm length travelling at half a body length per second. Percentage value at the end of the bar indicates the drag reduction compared to the carapace shape of the same species with the highest drag force. Note: forces for *I. auritus* morphs A and B, both 25 and 45 mm valves calculated using coefficients of drag and lift recovered for Reynolds numbers (Re) relating to a carapace length that gives a distance between the spines of 5 or 25 mm, respectively. Details of the model fit to simulated data given in the electronic supplementary material. Full results are provided in the electronic supplementary material, tables S1 and S2. (*b*) Maximum speeds for each carapace, calculated from the highest Reynolds number before flow transitioned to the turbulent regime, and the length of the fossil from which the carapace shape was collected, presented in carapace lengths per second and absolute values (mm s^−1^).

Drag coefficients were lowest at the highest Reynolds numbers for all shapes and were lower for shapes representing later stages in development for both species (figure 2). The transition from laminar to turbulent flow occurred at higher Reynolds numbers for shapes representing later stages in development for *I. auritus* (both morphs), but at the same Reynolds number (Re = 170) for *I. minor* (figure 3).

When carapace shapes were scaled to their length during development, the maximum velocity (in both ms^−1^ and body lengths s^−1^) was highest for the earliest stages in development, with the exception of morph A, where the 25 mm representative was outperformed by the 45 mm shape in ms^−1^ (figure 3). However, when scaled to the same length, the drag forces experienced by carapace shapes representing earlier stages of development were much higher than those representing later stages (electronic supplementary material, tables S1 and S2) and those of *I. auritus* were generally lower than *I. minor* with the exception of the earliest development stage of the former (figure 3). If *I. auritus* grew isometrically (no change in shape with growth), then the highest velocity it would have reached before the onset of turbulent flow would have been less than half achieved by morph B at 25 mm and around one-quarter of morph B at 45 mm (figure 3*b*).

Drag forces for *Isoxys* are over an order of magnitude higher for a 5 mm long carapace travelling at 5 body lengths s^−1^ than a 5 mm carapace travelling at 1 body length s^−1^ (Re = 70 and 13.5, respectively; figure 3; see full details in the electronic supplementary material, table S1), and greater for *I. minor* than *I. auritus* (figure 3; see full details in the electronic supplementary material, table S1). Carapaces representing a later stage in development led to lower drag forces than those at earlier stages for *I. auritus* morph A and *I. minor*, with very similar performance for *I. auritus* morph B at 25 and 45 mm shapes (figure 3). All *I. auritus* carapace shapes outperformed the juvenile 5 mm carapace substantially (figure 3).

Lift coefficients were highest for the largest Reynolds numbers for all shapes. Carapaces representing adult morphologies of *I. auritus* display higher, more favourable lift/drag ratios than those representing earlier stages in ontogeny, though the higher drag-induced lift forces (probably resulting from the higher drag coefficients) mean that for a given Reynolds number, the earliest stages in development generated the highest lift coefficient. The maximum lift coefficient and absolute values of lift were generated by the largest specimens representing *I. auritus* morph B. The drag forces were lowest for later stages in ontogeny, but very little separated *I. auritus* morph B 25 and 45 mm shapes (figure 3).

**Figure 4.**
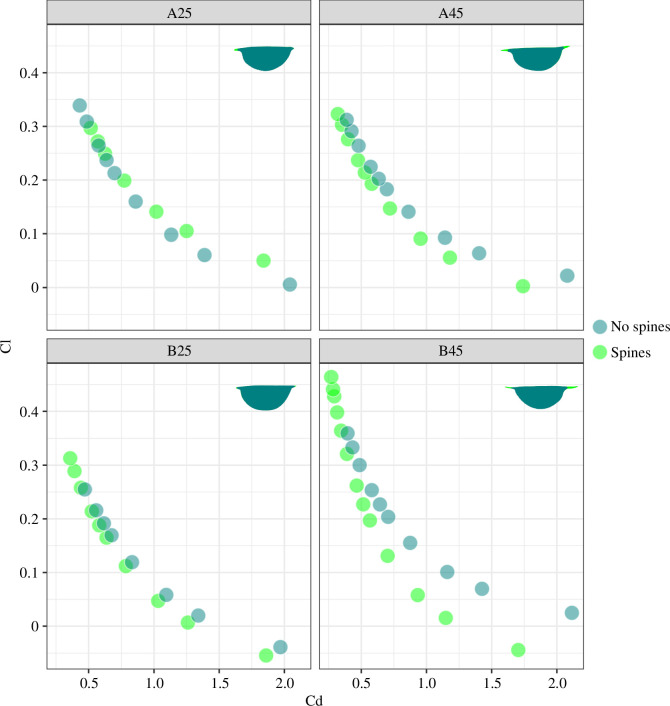
Plot of drag (Cd) and lift (Cl) coefficients for *I. auritus* morphs A and B, 25 and 45 mm carapace shapes, with spines and spines artificially removed. Outline used in the ANSYS simulations is indicated in the top right of each plot, with spines removed for ‘no spines’ analyses displayed in teal.

For the experiments isolating the impact of the spines, the lift coefficient for a given drag coefficient was higher for carapace shapes with the spines present rather than spines shortened, with the exception of the 25 mm long *I. auritus* morph A, where the removal of a very short anterior spine slightly decreased the drag coefficient for a given lift coefficient (figure 4). Both drag and lift forces were lower for carapaces with no spines when compared to their spined counterparts (when they were scaled so that the length of the carapaces between the spines was the same); however, the relative differences were much lower (within a few per cent) for drag forces than lift forces (5% for *I. auritus* morph B, 25 mm, >20% for all other carapace shapes; table 4).

**Table 4.** Drag (Cd) and lift (Cl) coefficients for simulations at Re = 170 and Re = 425, used to calculate drag and lift forces for carapaces with and without spines, scaled to be the same length when spines were removed. (Length of valve, including spines for relevant shapes, and swimming speed (velocity) used to calculate forces for each table row indicated.)

	Cd	Cl	valve length (m)	velocity (ms^−1^)	drag force (N)	drag force % change	lift force (N)	lift force % change
*I. auritus* morph A 25 mm carapace								
with spines	0.52	0.30	2.50 × 10^−2^	1.25 × 10^−2^	1.03 × 10^−3^	0.0	5.96 × 10^−4^	0.0
no spines	0.58	0.26	2.24 × 10^−2^	1.25 × 10^−2^	1.03 × 10^−3^	−0.1	4.74 × 10^−4^	−20.4
*I. auritus* morph A 45 mm carapace								
with spines	0.48	0.24	2.50 × 10^−2^	1.25 × 10^−2^	9.54 × 10^−4^	0.0	4.76 × 10^−4^	0.0
no spines	0.57	0.22	2.05 × 10^−2^	1.25 × 10^−2^	9.41 × 10^−4^	−1.3	3.70 × 10^−4^	−22.3
with spines	0.32	0.32	4.50 × 10^−2^	3.13 × 10^−2^	7.23 × 10^−3^	0.0	7.30 × 10^−3^	0.0
no spines	0.39	0.31	3.69 × 10^−2^	3.13 × 10^−2^	7.19 × 10^−3^	−0.6	5.78 × 10^−3^	−20.7
*I. auritus* morph B 25 mm carapace								
with spines	0.52	0.21	2.50 × 10^−2^	1.25 × 10^−2^	1.05 × 10^−3^	0.0	4.30 × 10^−4^	0.0
no spines	0.56	0.22	2.35 × 10^−2^	1.25 × 10^−2^	1.05 × 10^−3^	0.1	4.07 × 10^−4^	−5.2
*I. auritus* morph B 45 mm carapace								
with spines	0.47	0.26	2.50 × 10^−2^	1.25 × 10^−2^	9.34 × 10^−4^	0.0	5.26 × 10^−4^	0.0
no spines	0.58	0.25	1.95 × 10^−2^	1.25 × 10^−2^	9.09 × 10^−4^	−2.7	3.97 × 10^−4^	−24.5
with spines	0.32	0.40	4.50 × 10^−2^	3.13 × 10^−2^	7.12 × 10^−3^	0.0	8.99 × 10^−3^	0.0
no spines	0.39	0.36	3.51 × 10^−2^	3.13 × 10^−2^	6.96 × 10^−3^	−2.3	6.33 × 10^−3^	−29.6

## 4. Discussion

### 4.1. Relationship between ontogeny, size and hydrodynamic performance in *Isoxys*

The gradual change in shape coupled with the hydrodynamic improvement in carapaces representing later stages in development support an increasing role for streamlining and drag minimization for the carapace of *I. auritus* through ontogeny, in particular from the smallest size class of *I. auritus* to both morphs at 25 mm. This streamlining would have moderated the challenges of swimming at higher Reynolds numbers for larger *Isoxys* life stages [44]. Absolute drag forces were around 15% lower than if *I. auritus* had grown isometrically up to 25 mm in length rather than allometrically increasing asymmetry and spine length (figure 3), facilitating lower energy expenditure and faster swimming speeds. Notably, the performance of morph A and morph B for *I. auritus* indicate that the latter was able to swim faster before the onset of turbulent flow; however, the two morphs perform similarly in terms of absolute drag forces (figure 3). These differences may relate to the robustness of the morph A carapace. Morph A carapaces appear thicker than those of morph B, with compressed specimens showing no wrinkles and wide doublure and preserved in high relief [38]. *Isoxys minor* also shows a slight improvement in hydrodynamic performance through ontogeny, though this effect is smaller than for *I. auritus*. The small differences in the carapace outline and spine orientation of this species from the two deposits (Guanshan and Qingjiang) are not expected to lead to large differences in streamlining. This supports the previous interpretation that the carapace played a primary role in brooding eggs and protection and that streamlining was less important for *I. minor* [30]. In studies of extant isopods, differences in swimming speeds were greater than could be explained by differences in drag alone, indicating that body drag reduction correlates with other morphological attributes important for swimming efficiency such as muscle performance [45]. Indeed, swimming efficiency improves with body size in extant mysids [46]. While knowledge of the relative muscle power and swimming efficiency of *Isoxys* species is lacking, these comparisons to modern marine euarthropods suggest that *I. auritus* may have been a more powerful and more efficient swimmer than *I. minor* and that differences in the hydrodynamic performance of these two taxa might have been augmented by correlating differences in muscle power and swimming efficiency.

The increased streamlining of the carapace and elongation of the spines of *I. auritus* through ontogeny facilitated more efficient (lower drag) and faster (larger overall size, operation at a higher Reynolds number) swimming for this species compared to *I. minor*, or compared to the hypothetical of isometric rather than allometric growth for the species (figure 2). This suggests that allometry, specifically allometry improving hydrodynamic performance, was important for the evolution of larger sizes in *Isoxys*. The impact of the spines on drag was minimal (at most a few percentage points) compared to the changing shape of the carapace (figure 3 and table 4), but longer anterior spines did have a large impact on the lift forces—impacting it by 20−30% for adult carapaces (table 4). Thus, streamlining of the carapace outline between the spines, facilitated by allometric growth, impacted the drag coefficient more than the spines. This does not preclude the spines from also acting as an anti-predatory morphological feature; however, the increased length in later growth stages would imply that, for an anti-predatory feature, the function was more important for larger members of the species than smaller ones. Allometric growth of *Isoxys communis*, another relatively large *Isoxys* species, suggests that improvement of hydrodynamic performance was also important for larger sizes achieved by this species, as the valve length : height ratio and anterior spine length, both important properties impacting drag and lift [26], increased during growth [33].

Notably, the absolute speed (and speed in carapaces per second) before the onset of turbulent flow was highest for the earliest stages in development (with the smallest carapace sizes; figure 3*b*). This indicates that, for these early life stages, carapace shape did not provide a limit on swimming speed, and other aspects of the biology of the organisms such as muscle strength or appendage morphology were more important. Modern copepods and ostracods, which generally operate at Reynolds numbers from 1 to 50 (for animals up to 3 mm long), only swim rapidly (and at high Reynolds numbers) during escape reactions that last around 1 s [47], using setiose appendages as paddles to provide thrust [48] during normal swimming. At these lower Reynolds numbers, the (very minimal) possible hydrodynamic benefits of streamlined carapaces are outweighed by other considerations such as protection, while greater swimming speeds and endurance can be gained from setiose appendages providing greater thrust at these low Reynolds numbers. The smallest *I. auritus* reported from the Chengjiang display elongate appendages; however, these bear simple spines rather than setiose outgrowths [38], indicating that they were not the main source of thrust for these animals. Instead, *Isoxys* is thought to have swam using metachronal beating of appendages. This swimming mode uses cyclic, rather than synchronous, movement of appendages in a wave along the body to propel the animal forward [49]. Support for *Isoxys* using metachronal swimming comes from comparisons to swimming modes of extant crustaceans and the limited specialization of appendages along the body in *Isoxys* [31,32,35,39,50,51]. Further support comes from the ratio of the length of *Isoxys* appendage spacing to their length (the B : l ratio of [52]). This is close to 0.2 (as inferred from figures presented in [35]) and is at the lowest end for which metachronal swimming has been observed in modern marine animals [49,52]. In trilobites, for which metachronal swimming has also been inferred [53,54], favourable feeding currents have been suggested to occur during metachronal swimming [54]. However, it is unlikely that similar currents were used by *Isoxys* during feeding, as it probably fed in a raptorial fashion using anterior appendages [32,39,51].

At later life stages with larger carapace sizes and higher Reynolds numbers, streamlining of carapace shape became important for *Isoxys* species. In extant mysids, larger animals have either comparable or faster routine swimming speeds when compared to smaller ones [43,46], and this may have also been the case for *Isoxys* in the Cambrian oceans.

### 4.2. Predation, selection and a biotic push into the pelagic realm?

The focal taxa for this study, *I. auritus* and *I. minor*, most likely lived close to the seafloor and were not pelagic [26,30,38]. However, some *Isoxys* species were among the euarthropod pioneers in the pelagic realm, as demonstrated by the hydrodynamics and visual performance of a subset of species within the genus [26,31,34]. Comparison of *Isoxys* with closely related stem-group euarthropods (see phylogenies in [35]), including most probable sister taxa *Surusicaris* [55] and *Erratus* [56] indicates that the earliest diverging species within genus *Isoxys* probably had a symmetric carapace with short spines and that those pelagic taxa bearing streamlined carapaces with longer spines are derived. Thus, just as for many other groups, pelagic *Isoxys* species emerged from benthic stock [27,36].

Two species of *Isoxys, Isoxys longissimus* and *Isoxys paradoxus*, show comparable hydrodynamic performance to carapaces of the pelagic *Gnathophausia zoea. Isoxys paradoxus* are known from as early as Cambrian Age 3, a time that saw the appearance of large swimming predators [57–59] and a step change in trophic complexity compared to the Ediacaran and Terreneuvian. Broken carapaces found in coprolites and guts from this time [58,60,61] demonstrate that *Isoxys* were being predated upon. Predation pressure may have provided the biotic push for these pelagic *Isoxys* species to enter the water column. Predation pressure is expected to lead to an escalatory response by prey animals, and/or coevolutionary arms races [62,63], such as skeletonization or strengthening and elaboration of existing skeletons [7,64–66], growth to a size refuge [67–69] or behaviours such as burrowing [21]. In the case of *Isoxys,* the recognition that drag reduction facilitates larger size, but that larger size, in turn, facilitates lift generation (through both more favourable lift : drag ratios but also additional swimming power provided by larger limbs and muscles), provides a link between large size, anti-predatory morphological features (elongate spines) and expansion of some *Isoxys* species into the water column. Support for this comes from the recognition that large size can in itself be a response to predation pressure, with animals reaching a size refuge where they are less likely to be targets for predators. In addition, for a mobile animal such as *Isoxys*, it would facilitate more efficient swimming, augmented with the improved hydrodynamic performance and swimming efficiency of larger growth stages [43,46]. Reduced drag would have provided an additional benefit beyond efficient swimming and faster swimming speeds, as it would have reduced the disruption to the flow around the animal and hence the ‘noise’ and detectability by predators [70].

Elongate spines and more slender, hydrodynamic, carapaces are expressed later in the development of early diverging species such as *I. auritus,* allowing ecological and eventually genetic isolation from ancestral species [35,38,71–73]. As these features are probably derived (rather than ancestral) for the genus, current evidence suggests that the larger, more hydrodynamic *Isoxys* species arose through peramorphosis. Conclusive demonstration that the more hydrodynamic carapaces and elongate spines are the result of heterochrony requires detailed descriptions of the ontogeny of both ancestral and descendant forms [72]. For *Isoxys*, this would require knowledge of the ontogeny of taxa such as *I. longissimus*, which is currently known from very few specimens [39], and well-resolved phylogenetic analyses including more *Isoxys* taxa in order to confirm the hypothesized ancestral and descendant forms discussed above. A phylogenetic analysis including more *Isoxys* taxa and achieving finer resolution than achieved thus far [35] is limited by our lack of knowledge on soft part anatomy for most species. However, it is notable that peramorphosis has been considered a prime factor in generating increases in body size, as peramorphic forms tend to be larger than their ancestors [73]. This raises the possibility that for *Isoxys*, peramorphosis might have facilitated not just large body size but also elongation of spines and a more streamlined carapace, allowing expansion into the hyperbenthic and then pelagic realm, adding a developmental pathway alongside the ecological forcing from predation pressure. This peramorphic route into the plankton complements another heterochronic shift, neoteny, that has been a long-established pathway to evolve a planktic mode of life [27,37].

Once established within the pelagic realm, these *Isoxys* species would still have had to contend with predators, and selection for anti-predatory features such as reduced drag, larger size and elongated spines would have continued. Furthermore, increased demands of swimming at higher Reynolds numbers [44] and possibly higher metabolic demands of a pelagic mode of life [74] provide additional benefits for streamlined, low-drag carapaces. Thus the extreme forms of *I. longissimus* and *I. paradoxus,* with elongate spines and asymmetric carapaces found on the margins of *Isoxys* morphospace [26], probably reflect further selection pressure within the pelagic realm.

In summary, hydrodynamic performance improved during ontogeny for two species of *Isoxys, I. auritus* and *I. minor*. Larger *Isoxys* of both species have more hydrodynamic carapaces and more elongate spines. Based on our current understanding of the phylogenetic relationships within the genus, these hydrodynamic features represent derived traits, indicating that this could be a peramorphic trend. These traits can also be interpreted as anti-predatory, indicating that pelagic species of *Isoxys* arose from benthic stock at least partly as a result of predation pressure in the early Cambrian.

## Data Availability

All R code, outlines, ANSYS projects and ANSYS results have been uploaded to the Open Science Framework [75]. Supplementary material is available online [76].
